# In-depth proteomic analysis of shell matrix proteins of *Pinctada fucata*

**DOI:** 10.1038/srep17269

**Published:** 2015-11-26

**Authors:** Chuang Liu, Shiguo Li, Jingjing Kong, Yangjia Liu, Tianpeng Wang, Liping Xie, Rongqing Zhang

**Affiliations:** 1 Institute of Marine Biotechnology, Collaborative Innovation Center of Deep Sea Biology, School of Life Sciences, Tsinghua University, Beijing 100084 China; 2 Tsinghua-Peking Joint Center for Life Sciences, School of Life Sciences, Tsinghua University, Beijing 100084 China

## Abstract

The shells of pearl oysters, *Pinctada fucata,* are composed of calcite and aragonite and possess remarkable mechanical properties. These shells are formed under the regulation of macromolecules, especially shell matrix proteins (SMPs). Identification of diverse SMPs will lay a foundation for understanding biomineralization process. Here, we identified 72 unique SMPs using liquid chromatography-tandem mass spectrometry (LC-MS/MS) analysis of proteins extracted from the shells of *P. fucata* combined with a draft genome. Of 72 SMPs, 17 SMPs are related to both the prismatic and nacreous layers. Moreover, according to the diverse domains found in the SMPs, we hypothesize that in addition to controlling CaCO_3_ crystallization and crystal organization, these proteins may potentially regulate the extracellular microenvironment and communicate between cells and the extracellular matrix (ECM). Immunohistological localization techniques identify the SMPs in the mantle, shells and synthetic calcite. Together, these proteomic data increase the repertoires of the shell matrix proteins in *P. fucata* and suggest that shell formation in *P. fucata* may involve tight regulation of cellular activities and the extracellular microenvironment.

Pearl oysters, *Pinctada fucata*, are one of the most important economical pearl production species in China and Japan and are also one of the best studied biomineralization models^1^. Their shells are composed of calcite as the outer prismatic layer and aragonite as the inner nacreous layer. The biomineralized products possess superior mechanical^2^ and biological properties^3^ compared to common calcium carbonate. The shells typically consist of 95% CaCO_3_ and approximately 5% organic macromolecules including proteins, polysaccharides, and lipids^1^. Specifically, shell matrix proteins (SMPs) play important roles in crystal nucleation, polymorphism, morphology, and organization of calcium carbonate crystallites during shell formation^4^.

Since the cloning of the first SMP, Nacrein from *P. fucata* in 1996^5^, MSI60^6^, N16^7^, Prismalin-14^8^, Shematrin^9^, lysine(K)-rich matrix protein (KRMP)^9^, Aspein^10^, Tyrosinase^11^, N40^12^, Pif177^13^, Prisilkin-39^14^, PfN23^15^, and PfN44^16^ have been cloned and characterized. SMPs have been found to possess several functionalities: 1) they facilitate calcite (Aspein^17^) or aragonite crystallization (N40^12^), 2) they act as framework proteins (Shematrin^18^ and Prisilkin-39^14^), and 3) they guide calcium carbonate assembly (N16^19^).

A comprehensive characterization of SMPs will offer an opportunity to better understand biomineralization processes and refine the current “chitin-silk fibroin gel proteins-acidic macromolecules” model^20^. To achieve this characterization, proteomics has proven to be useful in identifying SMPs in high-throughput ways and has previously been used in *Lottia gigantea*^21^, *Pinctada margaritifera*^22^, *Pinctada maxima*^22^, *Mytilus coruscus*^23^, *Acropora millepora*^24^, *Stylophora pistillata*^25^ and *Cepaea nemoralis*^26^. In the present study, we identified 72 SMPs from the shells of *P. fucata.* Ethylenediaminetetraacetic acid (EDTA)-extracted proteins were subjected to SDS-PAGE followed by liquid chromatography–mass spectrometry (LC-MS/MS) analysis. Raw data from the LC-MS/MS were directly interrogated against the proteome derived from the draft genome of *P. fucata*^27^. Proteins with mascot scores above 5.0 and at least two matched peptide fragments were considered to be valid and were analyzed by BLAST, SMART and InterProScan. In addition to controlling CaCO_3_ crystallization process proteins, proteomic analysis suggests that diverse SMPs of *P. fucata* contain extracellular matrix-(ECM) related proteins. Moreover, diverse domains were found, including carbonic anhydrase, Glyco_hydro_18, Cu2_monooxygen, chitin-binding, complement control protein, von Willebrand factor type A, epidermal growth factor-like, tissue inhibitor of metalloproteinase, and Laminin_G_2/3. Immunohistological experiments showed localization of SMPs in the mantle cells, shells and synthetic calcite. Real-time PCR validated some representative genes *in vivo*. Together, our results increase shell matrix proteins’ repertoires in *P. fucata* and may guide the further study of SMPs.

## Methods

All methods were carried out in accordance with the approved guidelines. All experimental protocols were approved by the Animal Experimental Ethics Committee of Tsinghua University, Beijing, China.

### Sample preparation

The adult pearl oyster, *Pinctada fucata* (with shells 5.5–6.5 cm in length and 30–40 g of wet weight and approximately 2 years of age) was obtained from the Zhanjiang Pearl Farm (Guangxi Province, China). In the laboratory, the oysters were maintained at approximately 20 °C in an aquarium that contained aerated artificial seawater at 3% salinity.

### Shell preparation and proteins extraction

Cleaned shells of *P. fucata* were immersed in 5% sodium hydroxide for 24 h and were subsequently rinsed in the distilled water to avoid possible contamination of soft tissues adhered to the inner surface of nacre. The two layers of shells, the outer prismatic layer and the inner nacreous layer, were separated mechanically by abrasion before air-drying. Their fragments were pulverized (30 g) and were then decalcified with 0.8 M ethylenediaminetetraacetic acid (EDTA, pH 8.0) for 60 h at 4 °C with continuous agitation. For extraction of the soluble matrix, the supernatant was collected by centrifugation at 13,000 rpm for 30min at 4 °C, and was then desalted by ultrafiltration (3 K). For extraction of the insoluble matrix, the above precipitation were thoroughly rinsed with water and were treated with denaturing solution (30 mM Tris-HCl, pH 8.0, 1% sodium dodecyl sulfate (SDS), 10 mM dithiothreitol) at 100 °C for 30 min. After a short centrifugation, the denatured samples were ready to be applied on 12% SDS-polyacrylamide gels. Proteins were stained with Coomassie Brilliant Blue and were quantified by a BCA assay kit (Pierce).

### Characterization

The morphologies of the cleaned shells were examined by scanning electron microscope (SEM) (FEI Quanta 200, 15 kV) after being sputter-coated with a thin layer of gold nanoparticles.

### Immunolocalization of SMPs

#### Primary antibody production

Rabbit polyclonal antibodies were produced by injecting mixed shell matrix proteins in New Zealand rabbits^16^.

#### Western blotting

Proteins were electrophoretically transferred to PVDF membranes (Millipore) using a Mini Trans-Blot^®^ (Bio-Rad). Then, the PVDF membranes were blocked with 5% skim milk and were incubated with primary antibody (1:4000) for 2 h. After washing with Tris-Buffered Saline with 0.05% Tween 20 (TBST) and incubating with HRP-conjugated goat anti-rabbit secondary antibody (1:10000, Huaxingbio Science, China), detection was performed using 3,3’-diaminobenzidine (DAB) solution (TIANGEN, China). A control experiment was performed without the first antibody step.

#### Immunolocalization on the shells

Immunogold-labeling assays were conducted as described by the literature^15^,^28^ with some modifications. The antibodies were used at dilutions of 1:200. Goat anti-rabbit antibodies coupled to 15 nm gold particles (1:100, Huaxingbio Science, China) were used as the secondary antibodies. A control experiment was performed without the first antibody step. For high-resolution imaging, samples were sputter-coated with carbon and were analyzed using a Hitachi-SU8010 SEM at backscatter mode.

#### Immunolocalization on the mantle cells

Deparaffinized 10 μm sections of the mantle tissues, previously fixed for 24 h in Davidson fixative, were permeabilized for 10 min in TBST. Tissues were then incubated for 1 h in saturating medium (1% BSA, TBS) at room temperature. Then, samples were incubated with the anti-matrix antibody (1:100) for 1 h in TBST–BSA 1% at room temperature (RT). After rinsing in saturating medium, samples were incubated for 2 h at RT with HRP-conjugated goat anti-rabbit secondary antibody (1:10000, Huaxingbio Science, China). Finally, samples were observed with a DM-4000B Leica microscope. A control experiment was performed without the first antibody step^22^.

### Proteomic analysis

Protein bands selected from ESMs and EISMs of the prismatic and nacreous layers were excised and completely destained by washing with 50 μL of 50 mM NH_4_HCO_3_/CH_3_CN (50/50) mixture for 30 min at 37 °C. Then, reduction was conducted with 50 μL of 10 mM DTT in 50 mM NH_4_HCO_3_ for 1 h at 57 °C, and alkylation was performed with 50 μL of 100 mM iodoacetamide (IAA) for 30 min at RT in the dark. The cut gels were dried in CH_3_CN and were treated with 0.4 μg trypsin (Proteomics grade, Sigma) in 10 μL of 50 mM NH_4_HCO_3_ for 12 h at 37 °C. The solution was treated with 50 μL of 1% formic acid at 30 °C for 30 min under agitation. The digests were then lyophilized and suspended in 30 μL of 0.1% trifluoroacetic acid (TFA) and 4% acetonitrile for LC–MS/MS analysis. Five μL of sample was injected into the LTQ Orbitrap Velos mass spectrometer with Dionex U-3000 Rapid Separation nano LC system (Thermo Scientific) for analysis. MS data were acquired automatically using Analyst QS 1.1 software (Applied Biosystems) following a MS survey scan over m/z 350–1500 at a resolution of 60,000 for full scan and 2, 000 for MS/MS measurements.

The LC-MS/MS data were searched against the *P. fucata* predicted protein database (http://marinegenomics.oist.jp/genomes) using a Mascot 2.1 search engine with carbamidoethylated cysteine as a fixed modification and oxidized methionine and tryptophan as variable modifications. The peptide MS and MS/MS tolerances were set to 0.5 Da. Finally, sequences with mascot scores of at least 5.0 and with at least two matched peptide fragments were considered valid.

### Nucleotide and amino acid sequences analysis

Identification of proteins from above was attempted using Blastp and tBlastn searches against NCBI database (http://blast.ncbi.nlm.nih.gov/Blast.cgi). The protein sequences were deposited in NCBI (Table S1).The theoretical mass, isoelectric point and amino acid composition of the proteins were computed using ProtParam from the EXPASY online server. Conserved domains were predicted using SMART (http://smart.embl-heidelberg.de/) and InterproScan (http://www.ebi.ac.uk/interpro/ search/sequence-search). IUPRED (http://iupred.enzim.hu/) was used to recognize disordered regions from the amino acid sequences of SMPs based on the estimated pairwise energy content. XSTREAM (http://jimcooperlab.mcdb.ucsb.edu/xstream/) was used to isolate proteins with tandem-arranged repeat units using the default settings.

### Quantification of nacre and prism transcripts by real-time PCR

Total RNA from the mantle tissue and muscle were extracted using TRIzol reagent (Life Technologies) according to the manufacturer's instructions. RNA integrity was checked by agarose gel analysis, and RNA concentrations were determined by NanoDrop 2000 (Thermo Scientific). RNA concentration was approximately 1000 ng·μL^−1^, and A260/A280 was above 1.90. Then, cDNA was prepared by reverse transcription-PCR of the total RNA with GoScript^TM^ Reverse Transcription System (Promega) following the manufacturer’s instructions. Real-time PCR was conducted to quantify gene expression levels, with β-actin as an internal reference due to its relatively stable expression in mantle cells^15^,^16^. A typical reaction mixture is: SYBR^®^ Premix Ex Taq^TM^ II(Takara) 12.5 μL, forward primer 0.5 μL (10 μM), reverse primer 0.5 μL (10 μM), cDNA template 0.5 μL and H_2_O 6 μL. PCR parameters were: 95 °C for 30 s (1 cycle); 95 °C for 5 s, 60 °C for 30 s (40 cycles), 72 °C for 5 s; 72 °C for 30 s (see Table S2 for primers). Dissociation curves were generated to determine product purity and amplification specificity. Relative gene expression levels were calculated using two reference genes by the delta-delta method, as follows: fold = 2^−[ΔCt sample – ΔCt calibrator]^ = 2^−ΔΔCt^. Here, the “ΔCt calibrator” represents the mean ΔCt values of β-actin (AF378128.1) in the corresponding tissues.

## Results and Discussion

### Protein composition in the prismatic and nacre matrix

**Figure 1:**
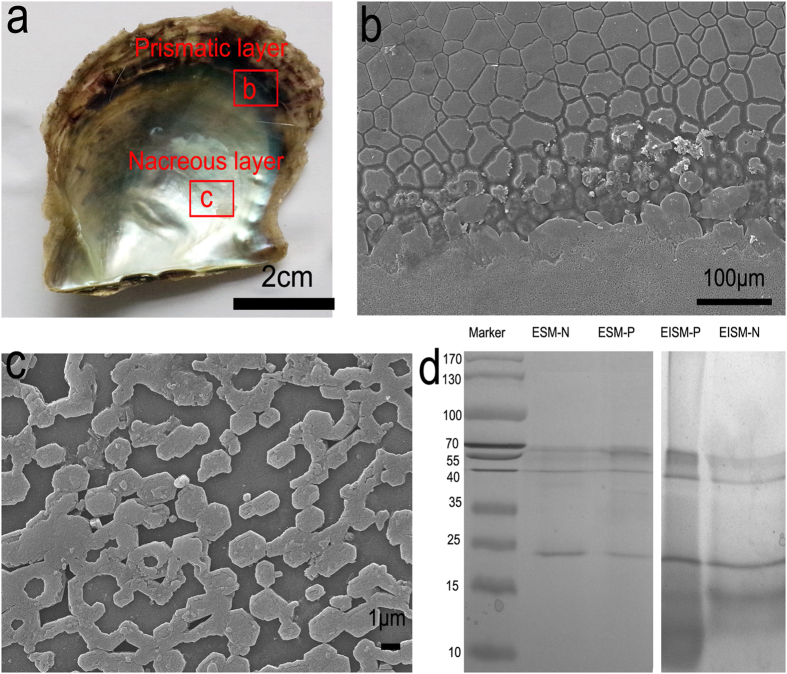
(**a**) Optical image shows the prismatic and nacreous layer of a typical shell (red box is examined by SEM), (**b**) SEM shows the shell surfaces of *Pinctada fucata*: the prismatic (**b**) and nacreous layer (**c**,**d**) SDS-PAGE of the four groups of extracted proteins (ESM and EISM are EDTA-soluble and EDTA-insoluble extracts, respectively; P and N mean the prismatic and nacreous layer, respectively).

The shell of *P. fucata* is composed of two layers, the prismatic layer and the nacreous layer (Fig. 1a). The prismatic layer is composed of prisms with length of 10–40 μm embedded in the organic sheath (Fig. 1b), and the nacreous layer is formed by stacked hexagonal nanotablets with side lengths of 0.5–3 μm (Fig. 1c). To extract SMPs, shells were first cleaned with NaOH to avoid possible contamination from outside organic matter. Then, separated shells, prism and nacre were dissolved with EDTA, leaving soluble and insoluble extracts. EDTA can chelate Ca^2+^, dissolve the shell and release the organic matrices. In this study, we observed that after 60 h, the shell can be fully dissolved. The yields of organic matrices from the shell were approximately 1.5–3.5 mg/g (determined by the concentration of proteins obtained from certain amounts of shell powders).

The soluble and insoluble extracts were subjected to SDS-PAGE (Fig. 1d). Protein bands in the gel were cut and digested with trypsin. Using LC-MS/MS, peptide fragments were searched against the proteome translated from the draft genome of *P. fucata*^27^. Consequently, through bioinformatics analysis such as BLAST, InterproScan and SMART, we identified 72 different SMPs out of 144 whole proteomes, in which 36 and 19 are solely found in the prismatic and the nacreous layers, respectively, while 17 are found in both layers. It should be noted that these identified proteins are related to the number of lysine and arginine residues available for trypsin cleavage in the protein sequences^29^. For example, Aspein and Prisilkin-39 lack trypsin cleavage sites, making them unsuitable for standard proteomic detection^29^. Jeana L. Drake *et al.* found thirty-six skeletal organic matrix proteins in the coral, *Stylophora pistillata*. Thirty-one were observed with tryptic digestion, while the remaining five were observed only after proteinase K digestion^25^. Therefore, the use of other digestion reagents only slightly increases the number of detected SMPs, and proteins found using trypsin as a digestion reagent likely represent most proteins in the shell. A typical process for LC-MS/MS analysis and protein identification is shown in Figure S1.

Intrinsic disorder (ID) refers to segments or to whole proteins that have no fixed 3D structures, with such disorder sometimes existing in the native state. ID domains are key molecular features that contribute to the formation and function of mollusk nacre; John Evans found that of 39 mollusk aragonite-associated protein sequences, 100% contain at least one region of intrinsic disorder or unfolding^30^. This researcher proposed that the intrinsically disordered domains are important for matrix assembly^30^. Hence, we used IUPRED to check the 35 unique SMPs found by proteomics. The results showed that 22 out of 35 sequences were predicted to have at least one region of intrinsic disorder (Table S3). Through XSTREAM, 7 out of 35 sequences were predicted to have tandem repeats (Table S3 and Figure S2). It is noteworthy that repetitive low complexity domains (RLCDs) are important but not the only implications for intrinsically disordered proteins.

According to the blastp results in the National Center for Biotechnology Information (NCBI) database, SMPs were divided into two groups: proteins with homology (e-value ≤ 10^−5^) (Table 1) and proteins without homology (e-value > 10^−5^) (Table 2). Compared to the shell proteomics of *Pinctada margaritifera*^22^, a closely related species with *P. fucata*, the numbers of proteins found in both layers were significantly improved. Mpn88, Nacrein, Nacrein-like, Shematrin-1, Shematrin-2, Shematrin-7, PTyr, PTyr1 and PNU1-9 were found in both layers, suggesting their potential roles in the formation of both layers in *P. fucata*. Conversely, the proteins in the two layers of *P. margaritifera* were Nacrein, nacre uncharacterized shell protein (NUSP18) and Shematrin 8, implying that the molecular toolkits responsible for formation of the prismatic and nacreous layers were extremely different. Sequence alignments from diverse mollusks and metazoans (Figure S3) showed that copper amine oxidase, peroxiredoxin, and chitinase were highly conserved across the metazoa. EGF domain-containing proteins and FN3 domain-containing proteins were highly conserved in the Pinctada family.

### Immunolocalization of proteins

**Figure 2:**
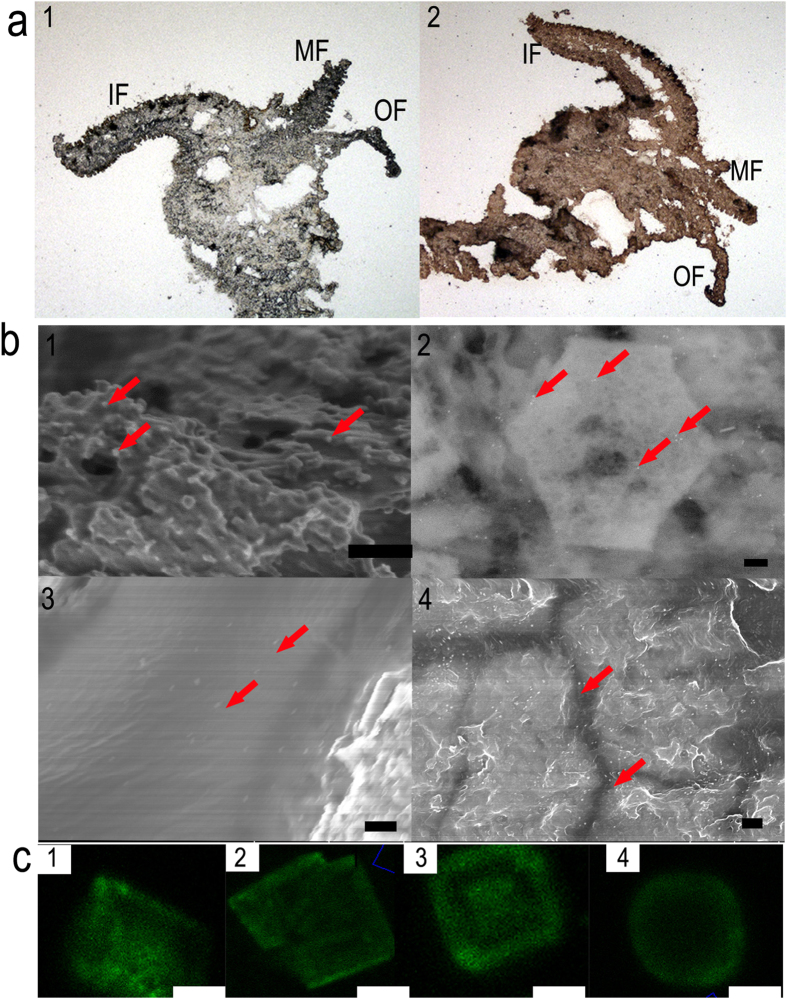
Immunolocalization of the shell matrix proteins (SMPs) of *P. fucata.* A polyclonal antibody raised against mixed extracted proteins is used to identify EDTA-soluble matrices (ESMs) and EDTA-insoluble matrices (EISMs). (**a**) Immunohistochemical localization in the mantle epithelia: control without the first antibody (a1); mantle sections with the first antibody (a2) (MF: middle fold; OF: outer fold; IF: inner fold); (**b**) Immunogold labeling of SMPs on the EDTA-mounted prismatic and nacreous layers. Layers with the first antibody (b1-b4). b1, b3 are prismatic layers and b2, b4 are nacreous layers. The red arrowheads indicate gold nanoparticles. (Scale bars, 200 nm) (**c**) Confocal fluorescence laser scanning microscopy images of SMPs *in vitro* show synthetic calcite after immunolabeling. Calcite in the presence of 1 μg·mL^−1^ ESM-P (c1), EISM-P (c2), ESM-N (c3), EISM-N (c4). (Microscopy settings are identical. The control is shown in Figure S6. Scale bars, 10 μm.)

To further validate the SMPs *in vivo* and *in vitro*, immunolocalization experiments were performed. Western blotting using polyclonal antibodies raised against the mixed shell matrix proteins of *P. fucata* showed ETDA-soluble matrices (ESMs) and ETDA-insoluble matrices (EISMs) all reacted with the antibodies (Figure S4). Immunohistochemical results clearly indicated that the SMPs are located in the mantle pallial and the mantle edge (Fig. 2a1) but showed no signal in the control (Fig. 2a2). Immunogold observations of the prismatic layer revealed that the antibodies exhibited in both the prismatic tablets (Fig. 2b1) and the chitin layer (Fig. 2b3). In the nacreous layer, antibodies exhibited a specific signal on nacre and localized in the interlamellar matrix that separated nacre tablets (Fig. 2b4) and in the nacre tablets (Fig. 2b2). In contrast, the control showed no gold nanoparticle signal (Figure S5). Immunolabeling synthetic calcite was conducted to verify the influence of extracted proteins on the growth of CaCO_3_. In the control group without the addition of extracted proteins, no fluorescence signal was observed under the same microscopy settings (Figure S6). By contrast, all four groups with the addition of extracted proteins exhibited fluorescence signals, indicating they could be occluded in/on the CaCO_3_. Specifically, SMPs from prismatic layers at approximately 1 μg·mL^−1^ had no noticeable effect on the morphology of CaCO_3_ and were evenly distributed (Fig. 2c1,c2). EDTA-soluble matrix from nacreous layers was concentrated in the center of crystals (Fig. 2c3). EDTA-insoluble matrix from nacreous layers changed the rhombohedral crystals into 5–10 μm rounded particles. In addition, the fluorescence intensity seemed to be concentrated at the edge of particles (Fig. 2c4). These results show that SMPs originate from the mantle cells and are finally embedded in the shells. In addition, SMPs can affect the CaCO_3_ crystallization process. Extensive studies have shown that SMPs, a single protein or mixed proteins can affect the nucleation, polymorphism and morphology of CaCO_3_^19^,^31^. The immune assay using antibodies against the SMPs suggests that SMPs from different parts of shells execute their distinct roles in CaCO_3_ crystallization, resulting in being occluded in/on CaCO_3_ with different patterns. The *in vitro* crystallization experiments were conducted under a basic condition (pH 8.0), which is close to the pH of seawater (8.2); therefore, the experiment may provide clues to the mechanism behind *in vivo* mineralization.

### Verification and quantification of matrix genes by real-time PCR

**Figure 3:**
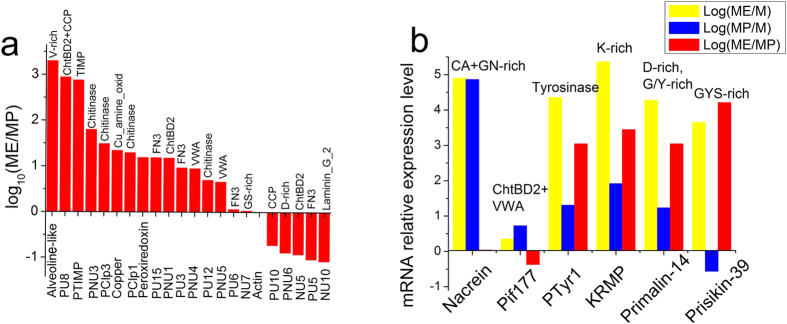
Real-time PCR of selected SMPs with domains or repeats shows relative gene expression in the mantle edge and mantle pallial of *P. fucata*. (**a**) Relative gene expression of twenty-one selected SMPs in the mantle edge compared with mantle pallial. Copper = Copper amine oxidase (**b**) Relative gene expression of six well-studied SMPs. (The longitudinal coordinates are the values of log_10_(ME/MP), ME: mantle edge, MP: mantle pallial)

To verify and quantify the proteins found by our proteomic analysis, real-time PCR was performed. As is known, the mantle edge is responsible for the formation of the periostracum and the prismatic layer, whereas the mantle pallial enabled the formation of nacreous layer^22^. Therefore, we examined the relative gene expression of 21 selected genes, which correspond to the proteins found in the proteomic analysis, in the mantle edge and mantle pallial of *P. fucata* (Fig. 3a and Table S4). According to previous studies, six developmental stages have been described across the entire *P. fucata* life cycle, including descriptions of the fertilized egg, trochophore stage, D-shaped stage, umbonal stage, juvenile, and adult^32^. The calcium carbonate crystal polymorphisms, the shell layer structure and the expression of SMPs change during these stages^32^. Almost all SMPs showed a dramatic increase at the adult stage. For example, the expression level of Pif and Prisilkin-39 in the adult stage is 2116.9 and 119.48 times that of the juvenile stage, respectively^32^. Hence, in the present study, all RNA was extracted from the mantle of adult oysters. SMPs encoded by the twenty-one genes are one valine (V)-rich protein (Alveoline-like protein), one glycine and serine (GS)-rich protein (NU7), one aspartic acid (D)-rich protein (PNU6), one tissue inhibitor of metalloproteinase (PTIMP), one Peroxiredoxin, one Copper amine oxidase, two Complement control protein (CCP) proteins (PU8 and PU10), one Laminin G protein (NU10), two von Willebrand factor type A (vWA) proteins (PNU4 and PNU5), three chitin-binding proteins (PU8, PNU1 and NU5), four chitinase (Clp1, Clp3, PNU3 and PU12) and four fibronectin type III (FN3) proteins (PU3, PU5, PU6 and PU15). Among all 21 tested genes, 18 genes (*Alveoline-like*, *PU8*, *PTIMP*, *PNU3*, *Clp3*, *Copper amine oxidase*, *Clp1, Peroxiredoxin*, *PU15*, *PNU1*, *PU3*, *PU12*, *PU6*, *PU10*, *PNU6*, *NU5*, *PU5* and *NU10*) were highly expressed in the mantle edge, mantle pallial, or both referenced to the muscle (Table S4), indicating they were likely to be involved in biomineralization process. *PNU4*, *PNU5* and *NU7* genes were neither highly expressed in mantle edge nor mantle pallial, so they may originate from other cells such as hemocytes^33^ and ultimately be involved in biomineralization^34^. Log(ME/MP) is the relative expression in the mantle edge to the mantle pallial, indicating roles of genes in the formation of the prismatic or nacreous layers. The results showed that most proteins found in the prismatic layer were highly expressed in the mantle edge (Fig. 3a). Surprisingly, PU10 and PU5 were found in the prismatic layer, but the corresponding genes were more highly expressed in the mantle pallial (Fig. 3a), suggesting their additional roles in nacreous layer formation. Similarly, previous studies showed that shematrin 5, a prism-related SMP, had much greater expression in the mantle pallial than in the mantle edge^35^. Additionally, six well-studied genes were also examined by real-time PCR (Figure 3b). *Nacrein* and *Pif177* genes are related to the formation of the nacreous layer and showed high expression in the mantle pallial (~10^5^ and ~10-fold referenced to the muscle, respectively). *Tyrosinase-1*(*Tyr-1*), *KRMP*, *Primalin-14* and *Prisikin-39* are genes related to the formation of the prismatic layer and exhibited high expression in the mantle edge (~10^4^, ~10^5^, ~10^4^ and ~10^3^-fold referenced to the muscle, respectively). Therefore, these data confirm that these genes are expressed in the calcifying tissues, and the corresponding proteins are embedded in the shells of *P. fucata*.

### Putative functions of the SMPs based on domains

To gain insight into the functions of SMPs, analysis of their sequences and domains is required. SMPs involved in biomineralization have several distinct characteristics^25^: 1) they are enriched in some specific amino acids such as aspartic acid, glutamic acid, glycine and serine, 2) they have flexible secondary structures and repeated low complexity domains (RLCDs) and 3) they possess multiple modularity. From well-known SMPs, biomineralization-related proteins contained carbonic anhydrases, chitin-binding, aragonite-binding, vWA, and D-rich domains. According the blast results, some well-studied SMPs have been found in the shells, verifying the effectiveness of our method. In general, the Shematrin^18^ and Tyrosinase family^11^ were among the most abundant proteins in the prismatic layer, and others included Nacrein^5^, Chitinase-like protein 1 (Clp1)^22^, Clp3^22^, KRMP^9^, Alveoline-like protein^22^, Amylase (GenBank, AGN55420.1), Prismalin-14^8^, Glycine-rich protein 2-like (PGRP2)^36^, Tissue inhibitor of metalloproteinases (TIMPs)^37^, Liprin-α protein, PPP-10^38^, Mantle protein 10 (PFMG1)^39^, Mpn88^29^, cement-like protein (SGMP1)^22^, Actin (GenBank, ACD99707.1) and Copper amine oxidase^22^. Nacrein^5^, Pif177^13^, N16^7^, and N19^40^ were enriched in the nacreous layer, and others were methionine-rich nacre protein (MRNP)^41^, MSI80 (GenBank, BAL45933.1), MSI60^6^, Mpn88^29^, Actin (GenBank, ACD99707.1), Peroxiredoxin^22^ and Polyubiquitin^42^ (Table 1). The SMPs that have previously been thoroughly characterized will not be discussed in this work.

Moreover, we identified some domains that are considered to play significant roles in the formation of biominerals. These domains include Glyco_hydro_18, Cu2_monooxygen, Chitin-binding domain 2 (ChtBD2), Complement control protein (CCP/SUSHI), Epidermal growth factor-like (EGF), TIMP, and Laminin_G_2/3. Compared with domains found in *P. margaritifera*, Glyco_hydro_20, EF-hand and Kunitz-like domains were not found. Kunitz domains (InterPro accession number, IPR002223) are the active domains of proteins that inhibit the function of protein degrading enzymes. Notably, the proteins extracted from prismatic layers carried more diverse domains than those from nacreous layers. Based on the domains, the SMPs were classified into the following three groups:

#### (1) Proteins that potentially regulate the extracellular microenvironment: carbonic anhydrase, chitinase, chitin-binding proteins and TIMP

The microenvironment, the immediate small-scale environment of mantle cells, and organs responsible for the formation of shells, includes pH, framework (mostly chitin) and proteinase in the seawater. Therefore, the extracellular microenvironment is critical for shell formation. Three types of proteins are related to the microenvironment: carbonic anhydrase, chitin relevant proteins and proteinase.

Carbonic anhydrase is responsible for controlling pH by converting CO_2_ to HCO_3_^-^ and is found in Nacrein. Chitin is the major framework in which CaCO_3_ grows^20^. Four proteins (PClp1, PClp3, PNU3 and PU2) contain Glyco_hydro_18 domains (IPR001223), which belong to a family of glycoside hydrolases, hydrolyzing the glycosidic bond between carbohydrates or between a carbohydrate and a non-carbohydrate moiety. Specifically, chitinase hydrolyzes chitin oligosaccharides. Real-time PCR showed that mRNAs of *PClp1*, *PClp3*, *PNU3* had high expression levels in the mantle edge and mantle pallial, suggesting their critical roles in shell formation (Fig. 3a and Table S4). A very recent microarray study had shown that chitinases were highly expressed at the D-shaped stage of *P. fucata* when the shell were first formed^32^. In addition to chitinase, another domain related to chitin is chitin-binding domain_2 (IPR002557). This domain was found in four proteins (PNU1, PU8, Pif177 and NU5), suggesting that chitin-binding ability is required by both layers. *PNU1* and *PU8* had higher mRNA expression levels in the mantle edge and lower levels in the mantle pallial, and *N-U5* was the opposite. *Pif177*, an important gene in the formation of nacre, had higher mRNA expression levels in the mantle pallial and lower in the mantle edge (Fig. 3b). In complex seawater, all secreted SMPs faced degradation by microorganism proteinases. Action must be taken to address this problem. We found tissue inhibitors of metalloproteinase (PTIMP and PTIMP3), which may complex with extracellular matrix metalloproteinases such as collagenases and irreversibly inactivate them (IPR001820). Members of this family are commonly found in the extracellular regions of vertebrate species. It has been inferred that the function of TIMP in *P. martensii* on nacre formation is to inhibit matrix metalloproteinases (MMP) activity due to their capacity to degrade most components of the extracellular matrix^37^. Thus, the existence of TIMP may ensure a “safe” place for other SMPs to execute their functions by creating suitable MMP-to-TIMP ratios. In humans, inhibition of MMP activity occurs in a 1:1 stoichiometric relationship and an imbalanced MMP to TIMP ratio may lead to various diseases^43^. In *P. martensii*, knocking down of TIMP by RNA interference results in abnormal nacre formation. However, a “suitable” MMP-TIMP ratio in the nacre formation has yet unknown. In fact, water-soluble extracts from the nacre of *P. margaritifera* possessed proteinase inhibitory activity against proteinase K^44^. Moreover, TIMP is also found in the shells of *C. gigas*^45^ and *M. coruscus*^23^. However, other protease inhibitors, such as Kunitz-like protease inhibitor or WAP (Whey acidic protein) domains proteins^46^, are not found in the shells of *P. fucata*. There is also a possibility that the amount of proteinase inhibitors in the shell are too low to detect. The problem may be addressed by RNA-seq of mantle cells in the future.

#### (2) Extracellular matrix- (ECM) related proteins: fibronectin-related proteins, laminin proteins, EGF proteins and liprin-α proteins

The shells are constantly growing and can be repaired after injury, indicating the possibility of communication between cells in different systems of *P.fucata* and the extracellular matrix (ECM). Proteomic data from *C. gigas* indicated that oyster shell matrix was not formed simply by self-assembling silk-like proteins but by diverse proteins through complex assembly and modification processes that may involve hemocyte and exosomes^45^. Indeed, it has been discovered that except RNA transport, ECM-receptor proteins were the most abundant in shell proteins. In agreement with previous results, we identified four ECM-related domains (fibronectin-related, laminin, EGF proteins and liprin-α) in the SMPs that may mediate communication between cells and the extracellular matrix.

Five uncharacterized proteins from the prismatic layer, PU3, PU5, PU6, PU15 and PU16, contain fibronectin type III (FN3) repeat regions (Fig. 3a and Table S4). Strikingly, PU3, PU5 and PU6 are among the most expressed proteins in the mantle pallial (Table S4), although they are from the prismatic layer. PU3, PU5, PU6, PU15 are highly expressed in both mantle edge and mantle pallial, implying their vital roles in the formation of both layers. FN3 is an approximately 100 amino acid domain that contains different tandem repeats with binding sites for DNA, heparin and the cell surface. The majorities of proteins containing FN3 are involved in cell surface binding or are receptor protein tyrosine kinases or cytokine receptors (IPR003961). The beta-sandwich structure of FN3 closely resembles that of immunoglobulin domains^47^. Notably, in *C. gigas*, a gene coding for a fibronectin-like protein was highly expressed at the early developmental stage when larval shells are formed in unison with chitin synthase^45^. Proteins containing fibronectin have been found in the shells of various biomineralization species, including *P. margaritifera*^22^, *C. gigas*^45^, *M. coruscus*^23^, and *A. millepora*^24^, suggesting important but yet unknown roles.

PU8 and NU10 possess a Laminin_G (LG) domain, which is thought to mediate attachment, migration and organization of cells into tissues during embryonic development by interacting with other extracellular matrix components (IPR001791). This domain has approximately 180–200 residues and is found in many extracellular and receptor proteins. LG modules have been implicated in interactions with cellular receptors such us α_6_β_1_ integrins, sulfated carbohydrates and other extracellular ligands^48^. In shells, proteins containing Laminin_G domain are found in *C. gigas*^45^, *M. coruscus*^23^ and *L. gigantea*^21^.

PU12 contains two epidermal growth factor-like domains (EGF), which are commonly found in extracellular proteins (IPR000742). EGF exhibits six conserved cysteine residues linked through three sulfide bonds. These domains are related to the immune system, apoptosis and Ca^2+^ binding. In shells, EGF proteins are found in *P. margaritifera*^23^*, C. gigas*^45^, *M. coruscus*^23^ and *L. gigantea*^21^.

Another protein belonging to this category is PLiprin-α, a member of the leukocyte common antigen-related (LAR) protein tyrosine phosphatase-interacting protein family. This protein binds to the tyrosine phosphatase LAR and appears to localize LAR to cell focal adhesions. This interaction may regulate the disassembly of focal adhesion and thus help orchestrate cell-matrix interactions (IPR029515)^49^. However, this protein is only found in the shells of *C. gigas*^45^ and not in the other species.

Interestingly, FN3, Laminin_G, EGF and Liprin-α proteins are able to interact with integrins, which are transmembrane receptors for cell-cell and cell-ECM interactions^50^. Integrin was identified in the matrix proteins of *S. pistillata*, a biomineralization model of coral species^25^. Moreover, α_v_β_6_ integrin has been shown to be expressed by ameloblasts and plays a crucial role in regulating amelogenin deposition and enamel biomineralization^51^. These studies suggest the integrin has a strong relationship with biomineralizaiton. Indeed, through real-time PCR, gene expression of *Integrin* in the mantle of *P. fucata* is higher than in the gonad, foot, muscle and gill (Figure S7).

#### (3) Other proteins of interest

Except for the above two groups of proteins, some genes are important due to their high expression in the mantle. An acidic protein, four VwA proteins, three complement control protein (CCP) proteins, a V-rich protein, and two enzymes (Tyrosinase and Copper amine oxidase) are discussed.

PNU6 is an extremely acidic protein with pI of 3.51 and possesses poly(D)_52_. The unique primary sequence indicates its role in the CaCO_3_ formation, possibly anchoring Ca^2+^ through the polyD domain, increasing concentration of local Ca^2+^ and favoring calcite precipitation. The function of PNU6 may be similar to the polyD-containing protein Aspein, an unusually acidic matrix protein found in *P. fucata*^17^.

Protein-protein interactions are important for shell formation because framework proteins, acidic proteins and others must cooperate to fulfill the complex requirement of biomineralization. VwA domains (IPR002035) in extracellular eukaryotic proteins mediate adhesion *via* metal ion-dependent adhesion sites (MIDAS), which are found in Pif177, PNU4, PNU5 and PU4. Three out of four proteins are found in the two layers, implying the importance of protein-protein interaction in both layers. In shells, vWA proteins are found in *P. margaritifera*^22^, *C. gigas*^45^, *M. coruscus*^23^ and *L. gigantea*^21^.

The complement control protein (CCP) modules (also known as short consensus repeats SCRs or SUSHI repeats) contain approximately 60 amino acid residues. They exist in a wide variety of complement and adhesion protein^52^ and are found in PU4, PU8 and PU10. Some of the proteins in this group are responsible for the molecular basis of the blood group antigens, which are surface markers on the outside of the red blood cell membrane (IPR000436). The CCP proteins indicate a putative relationship between shells and the immune system. CCP proteins are found in the shells of *P. margaritifera*^22^, *C. gigas*^45^, *M. coruscus*^23^ and *S. pistillata*^25^.

Alveoline-like protein (Alv), a V-rich protein, has only been discovered in the other two related species *P. margaritifera* and *P. maxima*^22^. Real-time PCR shows that it is highly expressed (almost 10^3^–10^4^ times referenced to the muscle) in both the mantle edge and mantle pallial (Fig. 3a and Table S4), indicating its critical roles in the formation of both calcite and aragonite. However, the function of Alv in CaCO_3_ crystallization is poorly understood.

Tyrosinase is an oxidase that controls the production of melanin by hydroxylation of a monophenol to o-quinone (IPR002227). It is reported that 21 tyrosinase genes were found in the genome of *P. fucata*^53^. As expected, four tyrosinase proteins were identified in the prismatic layer, and two tyrosinase proteins were found in both layers. Tyrosinase is also found in the shells of *P. margaritifera*^22^, *C. gigas*^45^, and *M. coruscus*^23^. Although the specific roles of tyrosinase are unknown, it is deduced that this protein plays distinctive roles in melanogenesis in pigmented shells^11^. Additionally, a tyrosinase gene is potentially involved in larval shell biogenesis in *C. gigas*^54^.

Copper amine oxidase catalyzes the oxidation of a wide range of biogenic amines including neurotransmitters, histamine and xenobiotic amines (IPR000269). In eukaryotes, they have a broad range of functions including cell differentiation and growth, wound healing, detoxification and cell signaling. In *P. fucata*, copper amine oxidase is primarily expressed in the mantle edge and has lower levels in the mantle pallial. However, its role has not previously been investigated in mollusks. A previous study in eastern oysters showed that the amine metabolic process was enriched in SMPs by Gene Ontology enrichment analysis^34^. This protein has not been reported in other shells of mollusks.

### The indication of proteomics on the shell mineralization mechanism

From our proteomic findings, some unexpected proteins have been discovered, indicating the intricacy of biomineralizaion in pearl oyster. The increased SMPs offer a chance to refine the previously proposed “chitin-silk fibroin gel proteins-acidic macromolecules” model^20^. The biomineralization mechanism of nacre has been previously proposed to consist of the following four stages: (1) assembly of the matrix, (2) the first-formed mineral phase, (3) nucleation of individual aragonite tablets, and (4) growth of the tablets to form the mature tissue^20^. At the first stage, the matrix is formed by layers of β-chitin, with a gel comprising silk-like protein filling the space between. Using proteomics, we identified four chitinases in both layers of shells. Chitinase is an enzyme that catalyzes the hydrolysis of β-1,4-N-acetyl-d-glucosamine linkages in chitin polymers and oligomers. Interestingly, we did not find any chitin synthase in our proteomic analysis, although chitin synthase genes can be found in the genome of *P. fucata*^53^. These results indicate that chitin synthases are located in the cell or on the cell membrane, while chitinases are secreted to the outside of cell to reconstruct the chitin network. Chitin binding proteins are able to interact with both chitin and minerals. For example, Pif protein with chitin domains have been proven to play an important role in the association of the inorganic phase and polysaccharide template and in the controlled nucleation of the initial mineral phase^31^. *In vivo*, Pif proteins are suggested to be able to work with other proteins such as N16, contributing to the formation of the lamellar sheet of nacre^13^. Silk-like proteins are rich in Gly and Ala or just in Gly. Such proteins include MSI60, MSI80, Shematrins, PGRP2, PAmylase, KRMP4, SGMP1, NU5, and NU7. Silk-like proteins are found in both the prismatic and nacreous layers, indicating their importance. One function of silk-like proteins is to act as a mild inhibitor of mineralization^20^. An *in vitro* crystallization assay demonstrates that recombinant KRMP3 inhibits the precipitation of CaCO_3_, affects the crystal morphology of calcite and inhibits the growth of aragonite *in vitro*, and these results are almost entirely attributed to the lysine-rich region. The Gly/Tyr-rich region of KRMP3 has the capacity to bind chitin^55^. Then, at the second stage, the first-form mineral phase, which is usually composed of amorphous calcium carbonate (ACC), is formed. ACC has been considered the precursor of biominerals, which exist in a wide range of living organisms, including nacre^56^. Mollusks requires high concentrations of Ca^2+^ and CO_3_^2-^ from the seawater to form ACC. CO_3_^2-^ is concentrated by carbonic anhydrase such as Nacrein, and Ca^2+^ is concentrated by acidic proteins such as Pif^13^ and Aspein^17^. ACC is unstable compared to calcite and therefore needs to be stabilized by specialized macromolecules such as Pif^31^. Several acidic proteins are found in the shell, including PNU2 and PNU6. At the third stage, nucleation of individual tablets begins, requiring some nucleators. Asp-rich proteins are thought to play a role in this stage. The final stage is growth of the tablets to form the mature tissue, and the shape of the final biominerals is thought to be thermodynamically driven^57^. At this stage, SMPs are incorporated in to the final biominerals. It is noteworthy that some SMPs, such as Pif, play multiple roles and can function during several stages. Furthermore, copper amine oxidase, peroxiredoxin and tyrosinase could be related to modification of SMPs, contributing to the overall structure and mechanical properties of shells.

Although this model can largely explain biomineralization from a crystal growth perspective, many proteins including FN3 proteins, CCP proteins, EGF proteins found in our proteomic analysis are not relevant to crystal growth so far. Recently, studies show that eastern oyster, *Crassostrea virginica,* forms its shell through a series of coordinated events involving hemocyte cells and ECM^58^. In fact, primary mantle cell cultures of *P. fucata* are able to precipitate amorphous calcium carbonate *in vitro*, suggesting the ability of mantle cells to perform biomineralization and shell formation processes^59^. Therefore, it is reasonable to hypothesize that the shell formation process is related to ECM-related proteins secreted by the mantle cells. These ECM-related proteins are part of SMPs and may play multiple roles. In fact, Osteopontin, a highly expressed ECM-related protein in bone that is glycosylated and enriched in acidic residues, is involved in a number of cellular processes including immune response and apoptosis besides its main role in biomineralization^60^.

Although we are aware that domains do not necessarily represent the exact function of proteins, the results in the present study are able to guide the further study of the diverse shell matrix proteins, improving our understanding of biomineralization.

## Conclusion

Using a proteomic approach, we identified 72 unique shell matrix proteins (SMPs) in which thirty-six are associated with the prismatic layer and nineteen are associated with the nacreous layer, while seventeen are associated with both layers. Based on immunohistological localization, these proteins were confirmed in the mantles, shells and synthetic calcites. In addition to controlling the CaCO_3_ crystallization process, the shell matrix proteins potentially regulate the extracellular microenvironment and communication between cells and the extracellular matrix (ECM). Our results increase the knowledge of shell matrix proteins in pearl oysters and offer an opportunity to refine the conventional “chitin-silk fibroin gel proteins-acidic macromolecules” model.

## Additional Information

**How to cite this article**: Liu, C. *et al.* In-depth proteomic analysis of shell matrix proteins of *Pinctada* *fucata*. *Sci. Rep.* **5**, 17269; doi: 10.1038/srep17269 (2015).

## Supplementary Material

Supplementary Information

## Figures and Tables

**Table 1:** Shell matrix proteins from the shells of *P. fucata* with blast homology.

Shell layer	Protein ID	Protein name	Protein homology	Domain
P,N	pfu_aug1.0_7401.1_31382.t1	Mpn88	*pfu* Mpn88-lack7	–
P,N	pfu_aug1.0_33972.1_19513.t1	Nacrein	*pfu* Nacrein	CA
P,N	pfu_aug1.0_8238.1_17260.t1	Nacrein-like	*pfu* Nacrein-like	CA
P,N	pfu_aug1.0_24266.1_33421.t1	Shematrin-1	*pfu* Shematrin-1	GY-rich
P,N	pfu_aug1.0_114185.1_13709.t1	Shematrin-2	*pfu* Shematrin-2	GYV-rich
P,N	pfu_aug1.0_411.1_00293.t1	Shematrin-7	*pfu* Shematrin-7	GLS-rich
P,N	pfu_aug1.0_109634.1_42492.t1	PTyr	*pfu* PfTy (tyrosinase-like protein)	Tyrosinase
P,N	pfu_aug1.0_10251.1_39018.t1	PTyr1	*pfu* Tyrosinase-like protein 1	Tyrosinase
P	pfu_aug1.0_14219.1_03462.t1; pfu_aug1.0_3035.1_59110.t1	PFMG1	*pfu* Mantle protein 10 (PFMG1)	Complement component C1q
P	pfu_aug1.0_15935.1_54245.t1	Prismalin-14	*pfu* Prismalin-14	GY-rich, D-rich
P	pfu_aug1.0_89559.1_56770.t1	PGRP2	*pfu* Glycine-rich protein 2-like	G-rich
P	pfu_aug1.0_275.1_14939.t1	PAmylase	*pfu* Amylase	A-rich
P	pfu_aug1.0_5122.1_59706.t1	KRMP4	*pfu* KRMP4	K-rich
P	pfu_aug1.0_3950.1_30572.t1	Shematrin-5	*pfu* Shematrin-5	GLY-rich
P	pfu_aug1.0_3212.1_37533.t1	PTyr1.1	*pfu* Tyrosinase-like protein 1	Tyrosinase
P	pfu_aug1.0_12145.1_17832.t1	PTyrB1.1	*pmax* Tyrosinase B1.1	Tyrosinase
P	pfu_aug1.0_21093.1_62062.t1	PTyr2	*pmar* Tyrosinase-like protein 2	Tyrosinase
P	pfu_aug1.0_16905.1_25558.t1	PTyr2.1	*pmar* Tyrosinase 2	Tyrosinase
P	pfu_aug1.0_13989.1_32380.t1	PClp1	*pmar* Clp1	Glyco_hydro_18
P	pfu_aug1.0_10761.1_31980.t1	PClp3	*pmar* Clp3	Glyco_hydro_18
P	pfu_aug1.0_20170.1_11291.t1	PPP-10	*pfu* PPP-10	S,Y-rich
P	pfu_aug1.0_89.1_57967.t1	Alveoline-like protein (Alv)	*pmar* Alveoline-like protein	V-rich
P	pfu_aug1.0_5205.1_38088.t1	PCopper	*pmar* Copper amine oxidase	Cu_amine_oxid
P	pfu_aug1.0_1824.1_37129.t1	Pliprin-α	*Cgi* Liprin-alpha-1	SAM domain
P	pfu_aug1.0_3120.1_51891.t1	Beta-actin	*pfu* Beta-actin	actin
P	pfu_aug1.0_17386.1_69084.t1	SGMP1	*pfu* SGMP1 (cement-like protein)	S,G-rich
P	pfu_aug1.0_12300.1_32179.t1	PTIMP3	*pmar* TIMP3	TIMP
P	pfu_aug1.0_8833.1_02656.t1	PTIMP	–	TIMP
N	pfu_aug1.0_13237.1_61173.t1; pfu_aug1.0_220503.1_50183.t1	Pif177	*pfu* Pif177	vWA+ChtBD2
N	pfu_aug1.0_277.1_07463.t1	N16	*pfu* N16 (Pearlin)	–
N	pfu_aug1.0_16924.1_69026.t1; pfu_aug1.0_2495.1_22991.t1; pfu_aug1.0_2495.1_22993.t1; pfu_aug1.0_36504.1_12255.t1	N19	*pfu* N19	–
N	pfu_aug1.0_170.1_36333.t1	MSI80	*Pfu*MSI80	A-rich
N	pfu_aug1.0_5926.1_52657.t1	MSI60	*pfu* MSI60	A,D-rich
N	pfu_aug1.0_13405.1_25160.t1	MRNP	*pfu*MRNP	M-rich
N	pfu_aug1.0_3120.1_51891.t1	Actin	*pfu* Actin	Actin
N	pfu_aug1.0_6671.1_24057.t1	Peroxiredoxin	*pfu* Peroxiredoxin	–
N	pfu_aug1.0_579791.1_57830.t1	Polyubiquitin	*pfu* Polyubiquitin	Ubiquitin

**Table 2:** Shell matrix proteins from the shells of *P. fucata* without blast homology.

Shell layer	Protein ID	Protein name	Domain	pI	MW kDa
P, N	pfu_aug1.0_10759.1_31979.t1	PNU1	ChtBD2	5.69	98.2
P, N	pfu_aug1.0_14699.1_32469.t1	PNU2	D-rich	4.85	43.6
P, N	pfu_aug1.0_14887.1_32490.t1	PNU3	Glyco_hydro_18	6.07	33.8
P, N	pfu_aug1.0_17316.1_18451.t1	PNU4	vWA	4.84	7.12
P, N	pfu_aug1.0_287428.1_50307.t1	PNU5	vWA	8.96	17.1
P, N	pfu_aug1.0_4881.1_67066.t1	PNU6	D-rich	3.51	21.4
P, N	pfu_aug1.0_75094.1_56521.t1	PNU7	–	5.40	13.8
P, N	pfu_aug1.0_7598.1_38581.t1	PNU8	–	9.86	11.6
P, N	pfu_aug1.0_86755.1_42180.t1	PNU9	–	8.20	9.6
P	pfu_aug1.0_1065.1_36810.t1	PU1	Cu2_monooxygen	9.49	53.6
P	pfu_aug1.0_10761.1_31980.t1	PU2	Glyco_hydro_18	8.85	58.5
P	pfu_aug1.0_11437.1_03076.t1	PU3	Fibronectin type 3	5.82	43.2
P	pfu_aug1.0_12760.1_53855.t1	PU4	vWA+ CCP	7.74	45.0
P	pfu_aug1.0_13143.1_39453.t1	PU5	Fibronectin type 3	4.90	33.2
P	pfu_aug1.0_155246.1_57288.t1	PU6	Fibronectin type 3	9.81	16.0
P	pfu_aug1.0_164724.1_21334.t1	PU7	–	11.6	29.0
P	pfu_aug1.0_1843.1_37145.t1	PU8	ChtBD2+CCP+ Laminin_G_3	5.13	21.8
P	pfu_aug1.0_20998.1_69384.t1	PU9	–	5.14	9.3
P	pfu_aug1.0_24840.1_62334.t1	PU10	Zn^2+^ binding + CCP	4.71	75.3
P	pfu_aug1.0_289511.1_28801.t1	PU11	–	9.43	21.5
P	pfu_aug1.0_853.1_22356.t1	PU12	EGF	8.30	38.4
P	pfu_aug1.0_9037.1_60542.t1	PU13	–	7.89	9.1
P	pfu_aug1.0_9053.1_67966.t1	PU14	Q-rich	10.2	77.3
P	pfu_aug1.0_9430.1_38910.t1	PU15	Fibronectin type 3	5.12	18.1
P	pfu_aug1.0_154829.1_06667.t1	PU16	Fibronectin type 3	9.63	13.6
N	pfu_aug1.0_12674.1_61081.t1	NU1	–	4.82	5.28
N	pfu_aug1.0_18113.1_25698.t1	NU2	Zf-Tim10_DDP	8.25	70.1
N	pfu_aug1.0_2111.1_22872.t1	NU3	–	7.98	29.2
N	pfu_aug1.0_21286.1_69411.t1	NU4	–	8.15	17.1
N	pfu_aug1.0_2323.1_15782.t1	NU5	ChtBD2, GS-rich	4.69	184.3
N	pfu_aug1.0_23607.1_62238.t1	NU6	–	8.48	7.93
N	pfu_aug1.0_24634.1_54990.t1	NU7	-GSM-rich	11.79	35.4
N	pfu_aug1.0_28.1_57930.t1	NU8	–	8.76	54.8
N	pfu_aug1.0_4561.1_44973.t1	NU9	–	8.76	46.5
N	pfu_aug1.0_954.1_58317.t1	NU10	Laminin_G_2	9.62	17.8

Aberration: P=prismatic layer, N=nacreous layer; ‘P, N’ means that the SMPs were found in both the layers; *Pfu=Pinctada fucata*, *Pmar*=*Pinctada margaritifera*, *Pmax=Pinctada maxima*, *Cgi= Crassostrea gigas*; CA=Carbonic anhdyrase; EGF=Epidermal growth factor-like; VWA= von Willebrand factor (vWF) type A domain; ChtBD2=Chitin binding domain 2; CCP/SCR/SUSHI =The complement control protein; TIMP=Tissue inhibitor of metalloproteinase; SAM=sterile alpha motif; Zf-Tim10_DDP= Zinc finger (Znf) domain. “-” represents undetected. Notes: The protein ID is the gene ID in the draft genome of *Pinctada fucata,* which is available at http://marinegenomics.oist.jp/genomes/viewer?project_id=20&current_assembly_version=ver1.0.

## References

[b1] Simkiss K. & Wilbur K. M. Biomineralization, (Elsevier, 2012).

[b2] Ling Li & Ortiz C. Pervasive nanoscale deformation twinning as a catalyst for eficient energy dissipation in a bioceramic armour. Nat. Mater. 13, 1–7 (2014). 24681646 10.1038/nmat3920

[b3] Kim H. *et al.* The role of nacreous factors in preventing osteoporotic bone loss through both osteoblast activation and osteoclast inactivation. Biomaterials 33, 7489–7496 (2012). 22809648 10.1016/j.biomaterials.2012.06.098

[b4] Falini G., Albeck S., Weiner S. & Addadi L. Control of aragonite or calcite polymorphism by mollusk shell macromolecules. Science 271, 67–69 (1996).

[b5] Miyamoto H. *et al.* A carbonic anhydrase from the nacreous layer in oyster pearls. Proc. Natl. Acad. Sci. USA 93, 9657–9660 (1996). 8790386 10.1073/pnas.93.18.9657 PMC38484

[b6] Sudo S. *et al.* Structures of mollusc shell framework proteins. Nature 387, 563–564 (1997). 9177341 10.1038/42391

[b7] Samata T. *et al.* A new matrix protein family related to the nacreous layer formation of *Pinctada fucata*. FEBS Lett. 462, 225–229 (1999). 10580124 10.1016/s0014-5793(99)01387-3

[b8] Suzuki M. *et al.* Characterization of Prismalin-14, a novel matrix protein from the prismatic layer of the Japanese pearl oyster (*Pinctada fucata*). Biochem. J. 382, 205–213 (2004). 15132736 10.1042/BJ20040319 PMC1133932

[b9] Zhang C., Xie L. P., Huang J., Liu X. L. & Zhang R. Q. A novel matrix protein family participating in the prismatic layer framework formation of pearl oyster, *Pinctada fucata*. Biochem. Bioph. Res.Co. 344, 735–740 (2006). 10.1016/j.bbrc.2006.03.179 16630535

[b10] Tsukamoto D., Sarashina I. & Endo K. Structure and expression of an unusually acidic matrix protein of pearl oyster shells. Biochem. Bioph. Res.Co. 320, 1175–1180 (2004). 10.1016/j.bbrc.2004.06.072 15249213

[b11] Zhang C., Xie L., Huang J., Chen L. & Zhang R. A novel putative tyrosinase involved in periostracum formation from the pearl oyster *Pinctada fucata*. Biochem. Bioph. Res.Co. 342, 632–639 (2006). 10.1016/j.bbrc.2006.01.182 16488396

[b12] Yan Z. *et al.* N40, a novel nonacidic matrix protein from pearl oyster nacre, facilitates nucleation of aragonite *in vitro*. Biomacromolecules 8, 3597–3601 (2007). 17929965 10.1021/bm0701494

[b13] Suzuki M. *et al.* An acidic matrix protein, Pif, is a key macromolecule for nacre formation. Science 325, 1388–1390 (2009). 19679771 10.1126/science.1173793

[b14] Kong Y. *et al.* Cloning and characterization of Prisilkin-39, a novel matrix protein serving a dual role in the prismatic layer formation from the oyster *Pinctada fucata*. J. Biol. Chem. 284, 10841–10854 (2009). 19233851 10.1074/jbc.M808357200 PMC2667771

[b15] Fang D. *et al.* Novel basic protein, PfN23, functions as key macromolecule during nacre formation. J. Biol. Chem. 287, 15776–15785 (2012). 22416139 10.1074/jbc.M112.341594 PMC3346131

[b16] Pan C. *et al.* A novel acidic matrix protein, PfN44, stabilizes magnesium calcite to inhibit the crystallization of aragonite. J. Biol. Chem. 289, 2776–2787 (2014). 24302723 10.1074/jbc.M113.504027 PMC3908410

[b17] Takeuchi T., Sarashina I., Iijima M. & Endo K. *In vitro* regulation of CaCO_3_ crystal polymorphism by the highly acidic molluscan shell protein Aspein. FEBS Lett. 582, 591–596 (2008). 18242173 10.1016/j.febslet.2008.01.026

[b18] Yano M., Nagai K., Morimoto K. & Miyamoto H. Shematrin: A family of glycine-rich structural proteins in the shell of the pearl oyster *Pinctada fucata*. Comp. Biochem. Physiol. B 144, 254–262 (2006). 16626988 10.1016/j.cbpb.2006.03.004

[b19] Seto J. *et al.* Nacre protein sequence compartmentalizes mineral polymorphs in solution. Cryst. Growth Des. 14, 1501–1505 (2014).

[b20] Addadi L., Joester D., Nudelman F. & Weiner S. Mollusk shell formation: a source of new concepts for understanding biomineralization processes. Chem-Eur. J. 12, 980–987 (2006). 16315200 10.1002/chem.200500980

[b21] Marie B. *et al.* The shell-forming proteome of *Lottia gigantea* reveals both deep conservations and lineage-specific novelties. FEBS J. 280, 214–232 (2013). 23145877 10.1111/febs.12062

[b22] Marie B. *et al.* Different secretory repertoires control the biomineralization processes of prism and nacre deposition of the pearl oyster shell. Proc. Natl. Acad. Sci. USA 109, 20986–20991 (2012). 23213212 10.1073/pnas.1210552109 PMC3529032

[b23] Liao Z. *et al.* In-depth proteomic analysis of nacre, prism, and myostracum of Mytilus shell. J. Proteomics 122, 26–40 (2015). 25857279 10.1016/j.jprot.2015.03.027

[b24] Ramos-Silva P. *et al.* The skeletal proteome of the coral *Acropora millepora*: the evolution of calcification by co-option and domain shuffling. Mol. Biol. Evol. 30, 2099–2112 (2013). 23765379 10.1093/molbev/mst109 PMC3748352

[b25] Drake J. L. *et al.* Proteomic analysis of skeletal organic matrix from the stony coral *Stylophora pistillata*. Proc. Natl. Acad. Sci. USA 110, 3788–3793 (2013). 23431140 10.1073/pnas.1301419110 PMC3593878

[b26] Mann K. & Jackson D. J. Characterization of the pigmented shell-forming proteome of the common grove snail *Cepaea nemoralis*. BMC Genomics 15, 249 (2014). 24684722 10.1186/1471-2164-15-249 PMC4023409

[b27] Takeuchi T. *et al.* Draft genome of the pearl oyster *Pinctada fucata*: a platform for understanding bivalve biology. DNA Res., 19, 117–130 (2012). 22315334 10.1093/dnares/dss005 PMC3325083

[b28] Seto J., Zhang Y., Hamilton P. & Wilt F. The localization of occluded matrix proteins in calcareous spicules of sea urchin larvae. J. Struct. Biol. 148, 123–130 (2004). 15363792 10.1016/j.jsb.2004.04.001

[b29] Berland S. *et al.* Coupling proteomics and transcriptomics for the identification of novel and variant forms of mollusk shell proteins: a study with *P. margaritifera*. ChemBioChem 12, 950–961 (2011). 21404418 10.1002/cbic.201000667

[b30] Evans J. S. Aragonite-associated biomineralization proteins are disordered and contain interactive motifs. Bioinformatics 28, 3182–3185 (2012). 23060620 10.1093/bioinformatics/bts604

[b31] Bahn S. Y., Jo B. H., Hwang B. H., Choi Y. S. & Cha H. J. Role of Pif97 in nacre biomineralization: *in vitro* characterization of recombinant Pif97 as a framework protein for the association of organic–inorganic layers in nacre. Cryst. Growth Des. 15, 3666–3673 (2015).

[b32] Jun Liu *et al.* Microarray: A global analysis of biomineralization-related gene expression profiles during larval development in the pearl oyster, Pinctada fucata. BMC Genomics 16, 325 (2015). 25927556 10.1186/s12864-015-1524-2 PMC4445274

[b33] Mount A. S., Wheeler A., Paradkar R. P. & Snider D. Hemocyte-mediated shell mineralization in the eastern oyster. Science 304, 297–300 (2004). 15073378 10.1126/science.1090506

[b34] Wang X. *et al.* Oyster shell proteins originate from multiple organs and their probable transport pathway to the shell formation front. PLoS One 8, e66522 (2013). 23840499 10.1371/journal.pone.0066522 PMC3686672

[b35] Kinoshita S. *et al.* Deep sequencing of ESTs from nacreous and prismatic layer producing tissues and a screen for novel shell formation-related genes in the pearl oyster. PLoS One 6, e21238 (2011). 21731681 10.1371/journal.pone.0021238 PMC3120837

[b36] Funabara D. *et al.* Novel genes participating in the formation of prismatic and nacreous layers in the pearl oyster as revealed by their tissue distribution and RNA interference knockdown. PLoS One 9, e84706 (2014). 24454739 10.1371/journal.pone.0084706 PMC3893171

[b37] Yan F. *et al.* Tissue inhibitor of metalloproteinase gene from pearl oyster *Pinctada martensii* participates in nacre formation. Biochem. Bioph. Res.Co. 450, 300–305 (2014). 10.1016/j.bbrc.2014.05.118 24942875

[b38] Nakayama S. *et al.* Identification and characterization of a matrix protein (PPP-10) in the periostracum of the pearl oyster, *Pinctada fucata*. FEBS Open Bio 3, 421–427 (2013). 10.1016/j.fob.2013.10.001 PMC3821031 24251105

[b39] Liu H.-L. *et al.* Identification and characterization of a biomineralization related gene PFMG1 highly expressed in the mantle of *Pinctada fucata*. Biochemistry 46, 844–851 (2007). 17223706 10.1021/bi061881a

[b40] Yano M., Nagai K., Morimoto K. & Miyamoto H. A novel nacre protein N19 in the pearl oyster *Pinctada fucata*. Biochem. Bioph. Res.Co. 362, 158–163 (2007). 10.1016/j.bbrc.2007.07.172 17698035

[b41] Marie B. *et al.* Characterization of MRNP34, a novel methionine-rich nacre protein from the pearl oysters. Amino Acids 42, 2009–2017 (2012). 21590302 10.1007/s00726-011-0932-0

[b42] Fang D. *et al.* Ubiquitylation functions in the calcium carbonate biomineralization in the extracellular matrix. PLoS One 7, e35715 (2012). 22558208 10.1371/journal.pone.0035715 PMC3338455

[b43] Tency I. *et al.* Imbalances between matrix metalloproteinases (MMPs) and tissue inhibitor of metalloproteinases (TIMPs) in maternal serum during preterm labor. PLos One 7, e49042 (2012). 23145060 10.1371/journal.pone.0049042 PMC3493509

[b44] Bédouet L. *et al.* Heterogeneity of proteinase inhibitors in the water-soluble organic matrix from the oyster nacre. Mar. Biotechnol. 9, 437–449 (2007). 17393253 10.1007/s10126-007-7120-y

[b45] Zhang G. *et al.* The oyster genome reveals stress adaptation and complexity of shell formation. Nature 490, 49–54 (2012). 22992520 10.1038/nature11413

[b46] Marie B. *et al.* Proteomic analysis of the organic matrix of the abalone *Haliotis asinina* calcified shell. Proteome Sci. 8, 54 (2010). 21050442 10.1186/1477-5956-8-54 PMC2989941

[b47] Hytonen V. P. & Wehrle-Haller B. Protein conformation as a regulator of cell-matrix adhesion. Phys.Chem.Chem. Phys. 16, 6342–6357 (2014). 24469063 10.1039/c3cp54884h

[b48] Timpl R. *et al.* Structure and function of laminin LG modules. Matrix Biol. 19, 309–317 (2000). 10963991 10.1016/s0945-053x(00)00072-x

[b49] Serra-Pages C. *et al.* The LAR transmembrane protein tyrosine phosphatase and a coiled-coil LAR-interacting protein co-localize at focal adhesions. EMBO J. 14, 2827 (1995). 7796809 10.1002/j.1460-2075.1995.tb07282.x PMC398401

[b50] Humphries M. Integrin structure. Biochem. Soc.Trans. 28, 311–339 (2000). 10961914

[b51] Mohazab L. *et al.* Critical role for alphavbeta6 integrin in enamel biomineralization. J. Cell Sci. 126, 732–44 (2013). 23264742 10.1242/jcs.112599

[b52] Norman D. *et al.* Three-dimensional structure of a complement control protein module in solution. J. Mol. Biol. 219, 717–725 (1991). 1829116 10.1016/0022-2836(91)90666-t

[b53] Miyamoto H. *et al.* The diversity of shell matrix proteins: Genome-wide investigation of the pearl oyster, Pinctada fucata. Zool. Sci. 30, 801–816 (2013). 24125645 10.2108/zsj.30.801

[b54] Huan P., Liu G., Wang H. & Liu B. Identification of a tyrosinase gene potentially involved in early larval shell biogenesis of the Pacific oyster *Crassostrea gigas*. Dev. Genes Evol. 223, 389–394 (2013). 23897397 10.1007/s00427-013-0450-z

[b55] Liang J. *et al.* Dual roles of the lysine-rich matrix protein (KRMP)-3 in shell formation of pearl oyster, Pinctada fucata. PLos One 10, e0131868 (2015). 26161976 10.1371/journal.pone.0131868 PMC4498902

[b56] Nassif N. *et al.* Amorphous layer around aragonite platelets in nacre. Proc. Natl. Acad. Sci. USA 102, 12653–12655 (2005). 16129830 10.1073/pnas.0502577102 PMC1200266

[b57] Bayerlein B. *et al.* Self-similar mesostructure evolution of the growing mollusc shell reminiscent of thermodynamically driven grain growth. Nat. Mater. 13, 1102–1107 (2014). 25326825 10.1038/nmat4110

[b58] Johnstone M. B. *et al.* Cellular orchestrated biomineralization of crystalline composites on implant surfaces by the eastern oyster, *Crassostrea virginica* (Gmelin, 1791). J. Exp. Mar.Biol. Ecol. 463, 8–16 (2015).

[b59] Xiang L. *et al.* Amorphous calcium carbonate precipitation by cellular biomineralization in mantle cell cultures of *Pinctada fucata*. PLoS One 9, e113150 (2014). 25405357 10.1371/journal.pone.0113150 PMC4236139

[b60] Giachelli C. M. & Steitz S. Osteopontin: a versatile regulator of inflammation and biomineralization. Matrix Biol. 19, 615–622 (2000). 11102750 10.1016/s0945-053x(00)00108-6

