# Supplementary material for: In-depth proteomic analysis of shell matrix proteins of Pinctada fucata
Source: Sci Rep. 2015 Nov 26;5:17269. doi: 10.1038/srep17269 (PMC4660305; doi:10.1038/srep17269)
Supplement: Supplementary Information [file srep17269-s1.pdf]

Supplementary information

In-depth proteomic analysis of shell matrix proteins of *Pinctada fucata*

Chuang Liu[1,2], *Shiguo Li*[1], *Jingjing Kong*[1],

Yangjia Liu[1], *Tianpeng Wang*[1], *Liping Xie*[1]*, *Rongqing Zhang*[1]*

[1] Institute of Marine Biotechnology, Collaborative Innovation Center of Deep Sea Biology, School of Life Sciences, Tsinghua University, Beijing 100084 China,

E-mail: lpxie@mail.tsinghua.edu.cn (LX); rqzhanglab@mail.tsinghua.edu.cn (RZ)

[2] Tsinghua-Peking Joint Center for Life Sciences, School of Life Sciences, Tsinghua University, Beijing 100084 China

TableS1. Genes and their corresponding accession number in GenBank

Genes	GenBank Numbers
**NU1**	KP852316
**NU2**	KP852317
**NU3**	KP852318
**NU4**	KP852319
**NU5**	KP852320
**NU6**	KP852321
**NU7**	KP852322
**NU8**	KP852323
**NU9**	KP852324
**NU10**	KP852325
**PNU1**	KP852326
**PNU2**	KP852327
**PNU3**	KP852328
**PNU4**	KP852329
**PNU5**	KP852330
**PNU6**	KP852331
**PNU7**	KP852332
**PNU8**	KP852333
**PNU9**	KP852334
**PU1**	KP852335
**PU2**	KP852336
**PU3**	KP852337
**PU4**	KP852338
**PU5**	KP852339
**PU6**	KP852340
**PU7**	KP852341
**PU8**	KP852342
**PU9**	KP852343
**PU10**	KP852344
**PU11**	KP852345
**PU12**	KP852346
**PU13**	KP852347
**PU14**	KP852348
**PU15**	KP852349
**PU16**	KP852350
**PTIMP3**	KP852351
**PTIMP**	KP852352
**Peroxiredoxin**	KP852353
**PClp-1**	KP852354

**PClp-3**	KP852355
**Alveoline-like**	KP852356
**Pcopper**	KP852357
**Pliprin-alpha**	KP852358

Table S2. Primers used for real-time PCR

**Primers names**	**Sequence (5'-3')**
**PClp1-F**	CAGTGAAGCGTGGAGTGAAGTC
**PClp1-R**	ATCCCATCAAAGTCATGTAGCC
**PClp3-F**	CGATCCGTTTCTATGTACCCAC
**PClp3-R**	TTCGTAATCGCACTATTCGTTG
**Alveoline-F**	ATAGGTGTTGGCGGTCCATTAG
**Alveoline-R**	CGATTCACTCAGTCTGCGGATTA
**Copper-F**	AAGTAGGCGGTGCTAAAACATG
**Copper-R**	CGTGGAATACTGGTGGGTCGTT
**Peroxi-F**	GAGCCGAACGACCCACCAGTAT
**Peroxi-R**	GAAGCAATAGGCGCAAATCAGG
**PNU1-F**	ACTGCTGTTGTTGACGGTGAC
**PNU1-R**	GCTGAAGGCTATGATTTCTGTTG
**PNU3-F**	AGCGAAGATGTGGGTGCAGAG
**PNU3-R**	CGATGGTGGCATTTGGCTGAG
**PNU4-F**	GTTTACTTCTGACGGATGCTGC
**PNU4-R**	ATTCGCCAATGTTTACACGGT
**PU3-F**	GATGAGTATGGGCAACCTAAAG
**PU3-R**	CAGATTCAACAGACAGGCAGTG
**PU4-F**	CGAATGTCCAATACGCAACTT
**PU4-R**	GAGACTGACGAATAGGAACACG
**NU5-F**	CGGGTGCTTCACTGCCTATTT
**NU5-R**	ATTTCCATCGCTTGGCTTATC
**NU10-F**	CCAAGGTTGTCACAGCATCAG
**NU10-R**	CGTGGTTCCAAGTTACCTCCC
**NU7-F**	TGTCAGGTTTCAGCGTTGGTG
**NU7-R**	ATGCTGCGTTGGGATGTCTCG
**PTIMP-F**	ATGGCAGAACGGGATTTATTG
**PTIMP-R**	ACCCTTTATTCCACGCTCAAC

**PU12-F**	CACGGATCTTTCTGCTTTCAC
**PU12-R**	TGCATTTGCACTCCCCATTAT
**PU10-F**	ACTTGTGCTGAGAACACGGTAG
**PU10-R**	GACCTGGTTTAATTGTTGCTCTATC
**PU5-F**	TTTGGGTTGGGTCGGTTTTAT
**PU5-R**	AGGCGGATTCCATCTTATTGC
**PU6-F**	ACATACTACTTCCGTGCTTTTGC
**PU6-R**	GGAGCTGTCATATCTGTATCTTGC
**PU8-F**	TTGGTGCCCCTGATGAGTAAC
**PU8-R**	GACTTGCCAGTGTCCAGTTTAG
**PU15-F**	GTACATGGGTATCGGGCTTCA
**PU15-R**	ACTCCACTGCTCTGCAAAAGG
**PNU5-F**	GACTTTACCCAGCAGCTCATAG
**PNU5-R**	TCGGAACTGTAGACCACCATC
**PNU6-F**	AACCAAGCGGGTCAACCAAA
**PNU6-R**	GCATTTTCCCCACATTAGAGTT
**Actin-F**	CACAGCATTCATACAAGCAAAGG
**Actin-R**	TGGGGCATCGTCTCCTCCAAAC
**Nacrein-F**	GAGCCAGAGGATGGGGAAA
**Nacrein-R**	GCCTCCATAGGTGAAACGA
**KRMP-F**	TCACCCTTGGGATTGGAAATGCA
**KRMP-R**	GCCAAAGTTGTAATCATCGCCACC
**Pif177-F**	TGCTGCCATCACGTGAGTATG
**Pif177-R**	GACTTCCCTTTCTCACACTTCCA
**PTyr1-F**	ATTAGCCAATGCCATCAGGAG
**PTyr1-R**	ACCCGATGCCACCCTAAGATA
**Integrin1-F**	ACTACCGTTTACACGAACAGCC
**Integrin1-R**	CTCAAGGAGAACCGATGGATG

Table S3. Disorder region and tandem repeat in the SMPs

**Protein**	**Disorder region IUPRED**	**Tandem repeat**
**PNU1**	+	-
**PNU2**	+	+
**PNU3**	+	-
**PNU4**	+	-
**PNU5**	+	-
**PNU6**	+	-

**PNU7**	+	-
**PNU8**	-	-
**PNU9**	-	-
**PU1**	-	-
**PU2**	+	-
**PU3**	+	-
**PU4**	+	+
**PU5**	+	-
**PU6**	+	-
**PU7**	+	-
**PU8**	+	-
**PU9**	+	+
**PU10**	-	+
**PU11**	-	-
**PU12**	-	-
**PU13**	-	-
**PU14**	+	+
**PU15**	+	-
**PU16**	+	-
**NU1**	-	-
**NU2**	-	-
**NU3**	+	-
**NU4**	-	-
**NU5**	+	+
**NU6**	-	-
**NU7**	+	+
**NU8**	+	-
**NU9**	-	-
**NU10**	-	-

Table S4. Real time PCR data of selected genes show the relative gene expression in the mantle edge and pallial of *P. fucata*

**Gene**	**Protein/domain**	**Log (ME/MP)**	**Log (ME/M)**	**Log (MP/M)**
**21 SMPs selected from the proteome**				
Alveoline-like	Alveoline-like protein	3.29808	4.26334	0.96525
PU8	Chitin-binding and CCP	2.94362	2.8926	-0.05102
PTIMP	Tissue inhibitor of metalloproteinase	2.87856	3.41698	0.53842
PNU3	Chitinase	1.79911	2.84443	1.04533
Clp3	Chitinase-like 3	1.48995	3.54869	2.05874
Copper	Copper amine	1.34169	0.62118	-0.72052

	oxidase			
Clp1	Chitinase-like 1	1.29639	2.44617	1.14978
Peroxiredoxin	Peroxiredoxin	1.19118	1.4457	0.25452
PU15	1 FN3	1.18803	1.72517	0.53714
PNU1	Chitin-binding	1.17868	1.16589	-0.01279
PU3	3 FN3	0.9648	4.14413	3.17933
PNU4	VWA	0.95035	-0.00933	-0.95967
PU12	Chitinase	0.69841	1.84462	1.14621
PNU5	VWA	0.6576	-0.31639	-0.974
PU6	FN3	0.06161	2.18946	2.12785
NU7	G,S rich	0.02786	-0.49641	-0.52429
Actin	Actin	0	0	0
PU10	CCP	-0.71976	1.85905	1.42392
PNU6	D rich	-0.88336	1.17748	2.06085
NU5	Chitin-binding	-0.91768	-0.3796	0.53809
PU5	2 FN3	-1.03063	1.61431	2.64495
NU10	Laminin	-1.07043	0.54753	1.61795
**Known SMPs**				
Nacrein	CA; GN rich	0.03988	4.90141	4.86153
Pif177	Chitin-binding and VWA	-0.37006	0.35393	0.72399
PTyr1	Tyrosinase	3.04343	4.35212	1.30869
KRMP	K rich	3.44464	5.36257	1.91793
Prismalin-14	D, G, Y rich	3.04324	4.27457	1.23133
Prisilkin-39	GYS rich	4.20967	3.65044	-0.55923

>pfu_aug1.0_3035.1_59110.t1 PFMG1 protein
MLAVLLFSALVGTAFSQGYPGPGKDVKIVAHPKAVEHQEARHVHHRETVKV
PIDVPVPQIETRHVVKYKENRIFRPKPVVKEHVQIIEEKRPFPVDQHVVQKIPK
PVVEVVKKPVVVVVQNIHHHKKQVVGVPHVKTVAEVVPNVVHQKVTYPVGK
GGYAGGPCKKMKLFRSLRRNEIIFRGNEVIIRRNEIIFRGNEVIVYGNEKKFRG
NEIVFRRNEMIFRRNEIIFRGSFCPSRRRPCTIARKVLITEPPLRLKINPFRRLATS
ASLLIVLLLLDNRCK

Peptide sequences	MH+, Da	Charge	Xcorr
VPIDVPVPQIETR	1463.81	2	3.48
EHVQIIEEK	1125.58	2	2.58
RPFPVDQHVVQK	1449.80	2	3.68
IPKPVVEVVK	1107.71	2	2.15
KPVVVVVQNIHHHK	1534.89	2	4.23
TVAEVVPNVVHQK	1419.79	2	2.79

Figure S1. Representative LC-MS/MS analysis: The protein derived from pfu_aug1.0_3035.1_59110.t1, which is PFMG1 protein through BLAST. Signal peptide was

underlined and the matched MS/MS peptides were marked pink or green; the table lists the corresponding parameters of peptides.

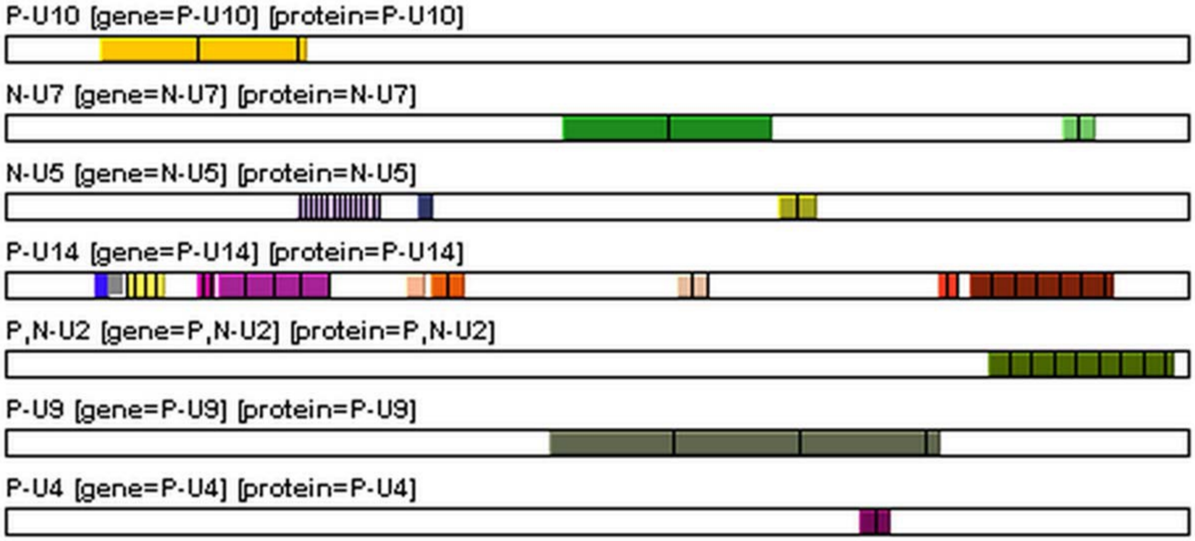

Figure S2. Tandem repeats in the SMPs predicted by XSTREAM

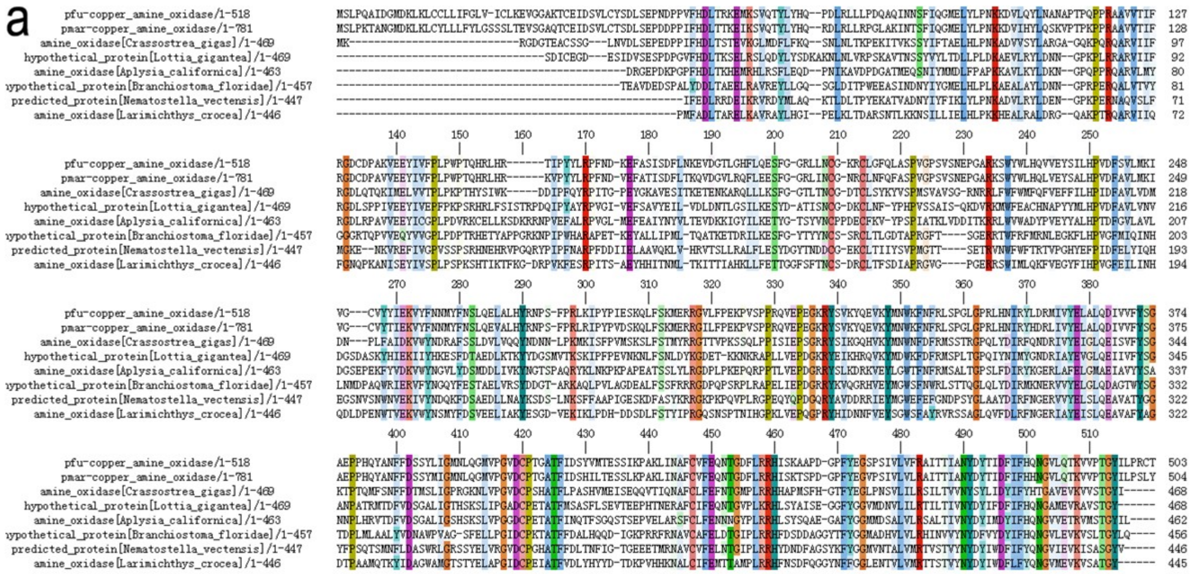

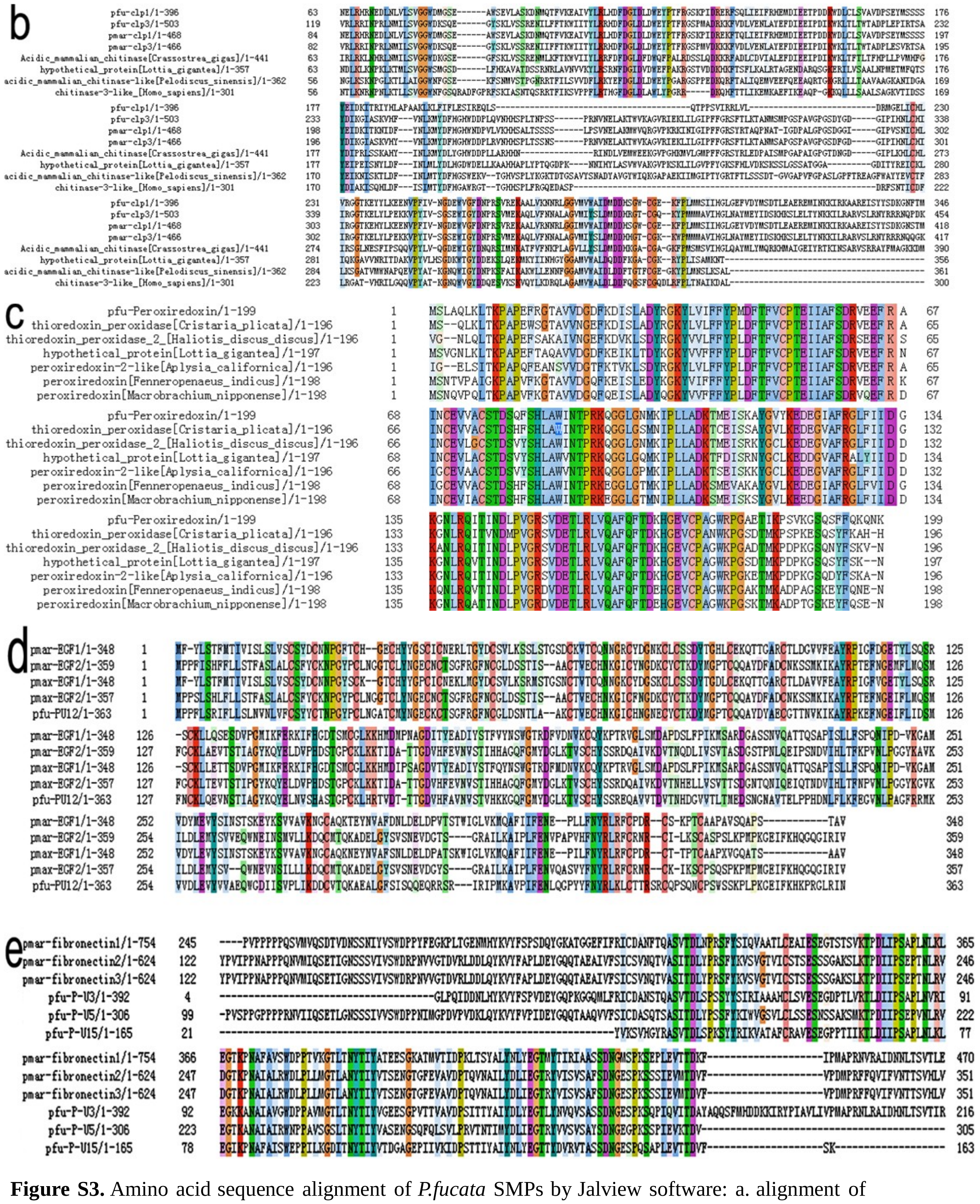

Figure S3. Amino acid sequence alignment of *P.fucata* SMPs by Jalview software: a. alignment of *P.fucata* copper amine oxidase b. alignment of *P.fucata* chitinase-like protein c. alignment of *P.fucata* peroxiredoxin d. alignment of *P.fucata* EGF domain containing protein PU12 e. alignment of *P.fucata* FN3 domain containing protein

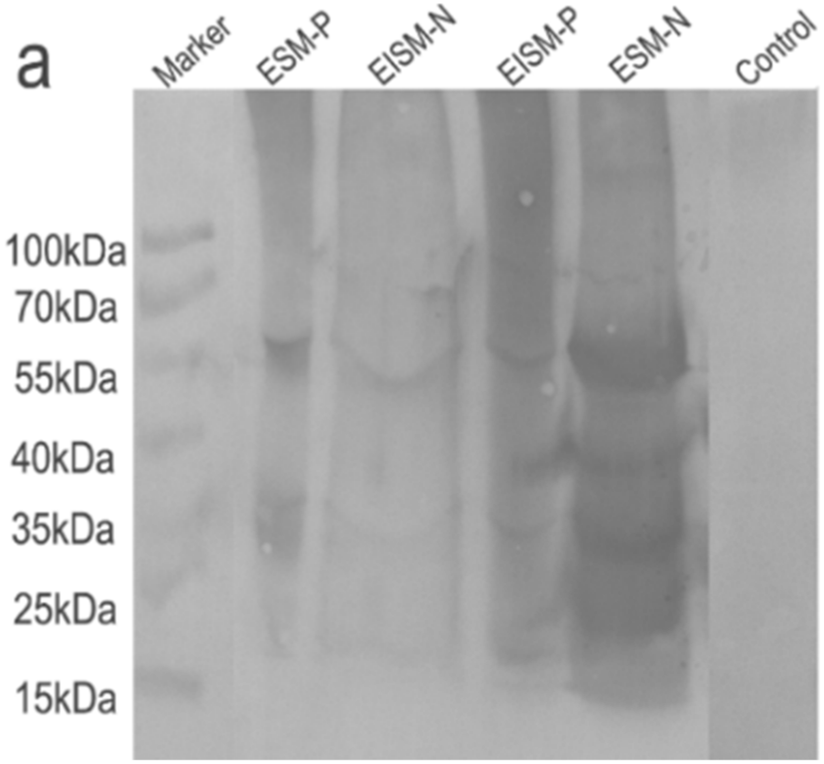

Figure S4. Western blot of EISM-P, N and ESM-P, N (N, nacre; P, prisms).

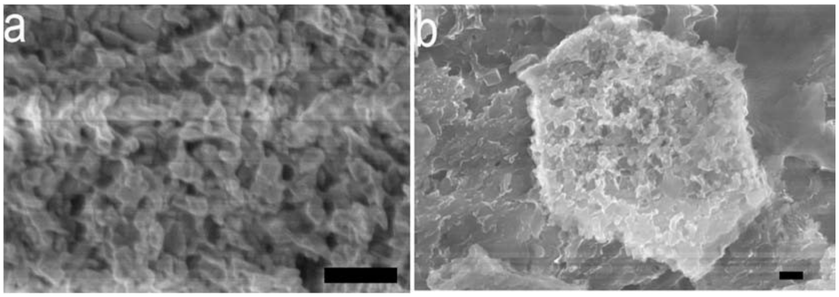

Figure S5. Immunogold labeling of matrix proteins on the EDTA mounted prismatic and nacreous layers without first antibody. a, prismatic layer and b nacreous layer. (Scale bars, 200nm)

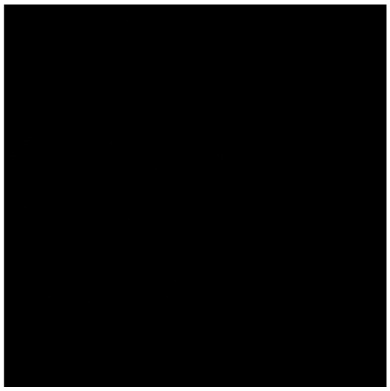

Figure S6. Confocal fluorescence laser scanning microscopy images of synthetic calcite without the addition of any extracted proteins. The image shows that the sample has no fluorescence signal.

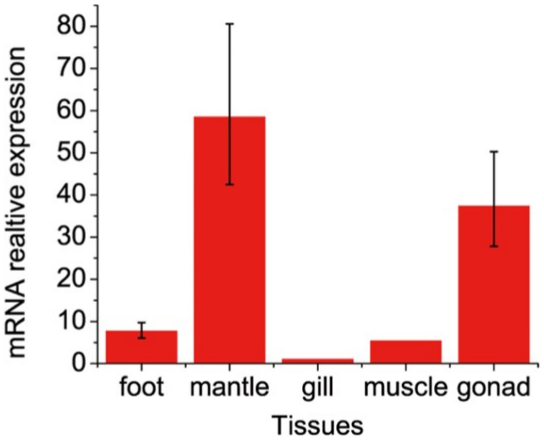

Figure S7. Tissue distribution of *Integrin* by real time RCR
